# Global interference during early visual processing: ERP evidence from a rapid global/local selective task

**DOI:** 10.3389/fpsyg.2013.00539

**Published:** 2013-08-27

**Authors:** Virginie Beaucousin, Grégory Simon, Mathieu Cassotti, Arlette Pineau, Olivier Houdé, Nicolas Poirel

**Affiliations:** ^1^Laboratoire de Psychopathologie et Neuropsychologie, EA 2027, Université Paris 8Paris, France; ^2^LaPsyDÉ, Unité CNRS 3521, Université Paris Descartes, Université de Caen, PRES Sorbonne Paris CitéParis, France; ^3^Institut Universitaire de FranceParis, France

**Keywords:** global processing, local processing, ERP, N1, global interference effect

## Abstract

Visual perception depends on the integration of local elements of a visual scene into a global frame. Evidence from behavioral studies shows that (1) the detection of the global frame is faster than the detection of the local parts, a phenomenon called the global advantage, and that (2) an interference of the global shape is also present during local processing. Together, these effects are called the global precedence effect (GPE). Even if the global advantage appears to impact neural processing as early as the first 100 ms post-stimulus, previous studies failed to find a global interference effect before 200 ms post-stimulus. Using for the first time a rapid display of letter component stimuli during a global/local selective task in which conditions with perceptual conflict, congruent and incongruent conditions were considered, the present event-related potential (ERP) study shows a global interference effect occurring as early as the time range of the N1 component. In particular, only congruent stimuli elicited similar N1 amplitude during the global and local tasks, whereas an increased of the N1 amplitude during the global task was observed (as compared to the local task) for both stimuli with perceptual conflict and incongruent stimuli. This finding corroborates the recent neural models of human visual perception.

## Introduction

When we go for a walk in a forest, do we perceive the forest first or the trees? The investigation of the mechanism of global visual perception has benefitted from Navon's work on global and local visual processing (Navon, 1977). He designed an elegant, now standard, paradigm using compound stimuli (for a review, see Navon, 2003). These stimuli are large letters (the global level) composed of small letters (the local level). When a participant is asked to identify the global or local level of a compound stimulus, a target stimulus is processed faster when participants are attending to the global, rather than the local level (i.e., the global advantage). Moreover, the presence of the non-target letter at the unattended global level slows down the detection of the target during local level processing (i.e., the global-to-local interference). Navon defined these effects as the “global precedence effect” (GPE) which was further illustrated by the famous statement that one sees “the forest before the trees” (Navon, 1977). Although the GPE can be reduced or even reversed by factors such as task variables (Shedden and Reid, 2001; Volberg and Hübner, 2007), the sparcity between the local features (Martin, 1979), the position of local elements and the saliency of the global form (Ripoll et al., 2005), the visual angle (Lamb and Robertson, 1990), the exposure duration (Andres and Fernandes, 2006) and the meaningfulness of the stimuli (Poirel et al., 2006, 2008), the dominance of global information is a robust effect (see Kimchi, 1992 and Hedgé, 2008, for a review).

Precious information regarding the timing of global/local perception has come from electrophysiological studies. The first stage that appears to be affected by the GPE corresponds to an event-related potential (ERP) peaking just before 100 ms after the stimulus presentation, namely the P1 component (Han et al., 1997, 1999, 2000a, 2001; Heinze et al., 1998; Yamaguchi et al., 2000). The P1 component is typically associated with perceptual analysis and this component could originate from extra-striate activations (Heinze et al., 1998). Rather than signaling a pure global advantage, P1 amplitude appears to be modulated by the visual complexity of stimuli (Johannes et al., 1995), hence the P1 amplitude increases as the visual complexity increases, i.e., as the number of local elements forming the global level increases (for a discussion, see Beaucousin et al., 2011). The global advantage appears rather to be reflected in the amplitude modulation of the following N1 component (Proverbio et al., 1998). Previous research has shown that the N1 component is sensitive to general visual discrimination processing (Vogel and Luck, 2000) and is specifically linked to selective attention of relevant elements required to perform a task (Mangun and Hillyard, 1991; Zani and Proverbio, 2012). The impact of the global advantage has been found in the early stages of perceptual processing; but to our knowledge, there has been no study that focuses directly on the global-to-local interference at these early stages.

However, previous studies have found an interference effect at the stages of the N2 component (Heinze and Munte, 1993; Han et al., 1997, 1999, 2001; Evans et al., 2000; Volberg and Hübner, 2004) and the P3 component (Ridderinkhof and van der Molen, 1995; Han et al., 1997; Volberg and Hübner, 2004). In agreement with previous results that hypothesized that an interference effect occurred as early as the N1 component (Beaucousin et al., 2011), one study has suggested that as early as N1, the consistency of the target with the information present at the other level can modulate its amplitude, but this effect did not survive planned comparisons (Han et al., 2000a). In addition, some procedures elicited the spread of attention (divided attentional task, Han et al., 2000a,b; hemifield presentation, Proverbio et al., 1998; Han et al., 1999; Evans et al., 2000) which has a great impact on the N1 amplitude (Mangun and Hillyard, 1988), and this could have prevented the detection of an early interference effect. Moreover, it is worth noting that several studies with compound letters stimuli did not include congruent compound stimuli in their paradigm (Evans et al., 2000; Yoshida et al., 2007) or include few congruent stimuli during detection task in which 50% (or even more) of trials have no target to detect (Heinze and Munte, 1993; Han et al., 1997, 1999, 2000a, 2001). However, a congruent situation, in which the same letter is presented at both global and local levels, represents an adequate baseline condition that corresponds more to real life situations (for example, the trees present at the local level are congruent with the global perception of the forest). Consequently, congruent situations seem useful in evaluating the strength of the interference effect produced in other global/local situations such as (1) the traditional “neutral” situation used in several studies, in which stimuli presented a non-target at the irrelevant level (e.g., Weissman et al., 2005; Yovel et al., 2005; Volberg and Hübner, 2007) and (2) incongruent situations with a response conflict, in which a different target letter is present at the irrelevant level. Importantly, it has been suggested that the “neutral” situation traditionally used in several studies could be also considered as conflicting situations (Poirel et al., 2006, 2008). Indeed, previous results evidenced that interference effect occurs as soon as different information are presented between global and local levels (Poirel et al., 2008, Experiment 2). In order to avoid any ambiguities we will consider stimuli presented a non-target at the irrelevant level—i.e., the traditional “neutral” stimuli mentioned above—as conflicting perceptual stimuli (see Weissman et al., 2003).

To investigate the potential interference of global information during local processing at early stages of visual perception, we used Navon's classic paradigm. Based on previous results, we have suggested that an early modulation of N1 amplitude is due to the global interference effect (Beaucousin et al., 2011). Thus, we hypothesize a modulation of the N1 amplitude between congruent and either conflicting perceptual conflict or incongruent compound stimuli rapidly presented during a global and local detection task. We propose that the amplitude of N1 will be smaller during local tasks than during global tasks for conflicting perceptual and incongruent stimuli because the global level tasks will automatically capture attentional resources during local processing, while such an amplitude difference will be reduced or even absent for congruent stimuli. Based on results from our previous experiment, we also hypothesize that this global-to-local interference effect will not affect the P1 amplitude because this component amplitude is rather modulated by the amount of visual complexity that is equivalent between the letter-based stimuli used in the present study (Johannes et al., 1995; Beaucousin et al., 2011).

## Materials and methods

### Participants

Twelve healthy volunteers (10 women) aged 23–37 years (29 ± 4 years, mean ± standard deviation) participated in the experiment. All participants were right-handed, based on the Edinburgh inventory (91 ± 11%, [100–71]; Oldfield, 1971). All participants had normal or corrected-to-normal vision. No participants reported neurological disorders or the use of psychoactive medications. All participants provided written informed consent in accordance with the Declaration of Helsinki (BMJ 1991; 302:1194).

### Stimuli and procedure

**Figure 1:**
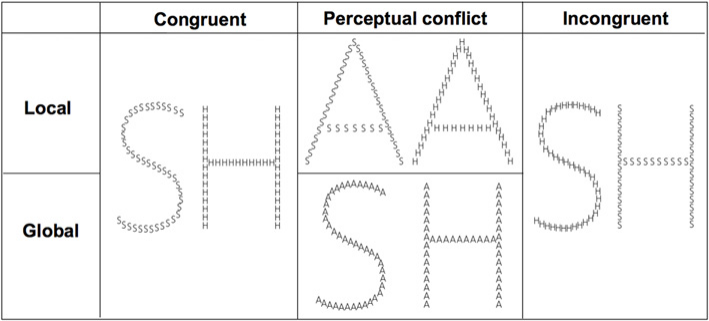
**Examples of stimuli**.

The compound stimuli consisted of a global letter always made up of 40 small local identical letters (Figure 1; see Poirel et al., 2008). Each of the small letters fit in virtual rectangles of 1.26 × 0.92° (height × width), while the virtual rectangle for the global letter subtends 8.5 × 11.4° of visual angle. The compound stimuli were based on combinations of “A” (non-target letter), “H” and “S” (target letters).

**Figure 2:**
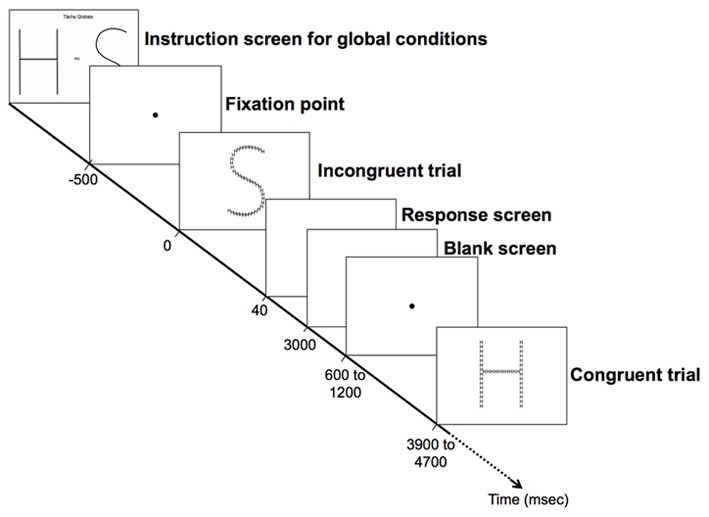
**Experimental design**. Each trial began with a central fixation point that lasted 500 ms. This fixation time was followed by the presentation of the compound stimuli, which was displayed for 40 ms on a black screen. The participants had to respond as quickly and accurately as possible by pressing a mouse button to report which of the two targets (“H” or “S”) they detected at the corresponding level. The response time (RT) was limited to 3000 ms (black screen). The inter-stimulus interval (ISI) was jittered between 600 and 1200 ms (black screen). All screens were black with white text or figure.

Participants were comfortably seated in front of a 19-inch screen linked to a laptop equipped with E-prime® 1.2 (Psychology Software Tools, www.pstnet.com). The stimuli were presented as white lines on a black background at a distance of ~72 cm. Prior to the experiment, the participants were trained with two blocks of trials that consisted of the different global and local selective-attention tasks of letter detection. The stimuli presented were either (1) congruent, with the same target letter, “H” or “S”, at both the global and local levels; (2) with a perceptual conflict, with a non-target letter “A” at the irrelevant level; (3) incongruent, with a different target at the global and the local levels (i.e., a global “H” comprised of local “S”, or a global “S” comprised of local “H”; see Figure 1). The order of the stimuli presentation in each block and the order of the blocks were randomized across participants. Prior to beginning of a block the participants viewed an instruction screen that indicated which level to consider (global or local; Figure 2). Then, the participants were asked to detect, as fast as possible, which of the two possible target letters (i.e., “H” or “S”) appeared at the target level while ignoring the other irrelevant level. Each trial began with a central fixation point that lasted 500 ms to minimize ocular movement. This fixation time was followed by the presentation of the compound stimuli, which was displayed for 40 ms on a black screen. The participants had to respond as quickly and accurately as possible by pressing a mouse button to report which of the two targets (“H” or “S”) they detected at the corresponding level: a left mouse button press indicated that an “H” was detected and a right mouse button press indicated that an “S” was detected. The response time (RT) was limited to 3000 ms. The inter-stimulus interval (ISI) was jittered between 600 and 1200 ms. The participants performed 8 blocks, including a total of 216 trials. Half of the blocks consisted of the global condition and the other half were the local condition (108 trials per experimental condition).

### ERP recordings

The electroencephalogram (EEG) was recorded from 32 sintered Ag/AgCl electrodes plugged into an electrode cap (EasyCap^©^, Herrsching-Breitbrunn, Germany). Scalp electrodes were placed according to the extended International 10/20 system. An electrode placed at AFz served as ground. A common reference method was used during acquisition and two linked earlobe electrodes were included in the recording montage for the off-line recalculation of the reference. Blinks and eye movements were monitored via two additional electrodes placed on the supraorbital ridge and on the outer canthus of the right eye. Electrode impedances were kept below 5 kΩ. The EEG was processed through a QuickAmp^©^ amplifier (www.brainproducts.com) set at a bandpass of 0.01–100 Hz, continuously digitized at 500 Hz.

The EEG was re-referenced off-line to the averaged left and right earlobes. ERPs were computed for epochs extending from 100 ms pre-stimulus to 1000 ms post-stimulus. Artifacts were reduced by eye movement correction using the regression-based approach (Gratton et al., 1983) and by rejection of trials with voltages ±150 μV in any EEG channel. Only 3.7% of the epochs were discarded due to excessive blinking (17 epochs, mean ± standard deviation = 1.4 ± 1.6; [max-min] = [5–0]) or incorrect responses (76 epochs across all the conditions, 6.3 ± 3.7; [12–1]). For the trials with correct responses, the mean ERPs were averaged for each subject and for each stimulus type.

### Statistical analysis

Reaction times (RTs) for correct responses were assessed using the general linear model (GLM) with two factors, condition (global or local task) and type of trial (congruent, with perceptual conflict, or incongruent).

ERP statistical analyses were performed on the mean amplitudes elicited during correct trials between 80 and 130 ms for P1 and between 150 and 220 ms for N1 from P7 and P8 electrodes that correspond to the maximal amplitude differences between conditions. P1 and N1 averaged amplitudes were analyzed using a GLM including three factors: condition (global or local task), type of trial (congruent, with perceptual conflict, or incongruent) and side (left or right hemi-scalp). All *post-hoc* comparisons were performed with Bonferroni tests. Note that only correct responses were included in the ERPs analyses to ensure that it will reflect the brain functioning that underlined the cognitive mechanisms involved during the processing of each type of trials. The factor of hemispheric side was included because it has been showed that the two hemispheres could present different sensibilities to global and local processing (Fink et al., 1996).

## Results

### Behavioral results

**Figure 3:**
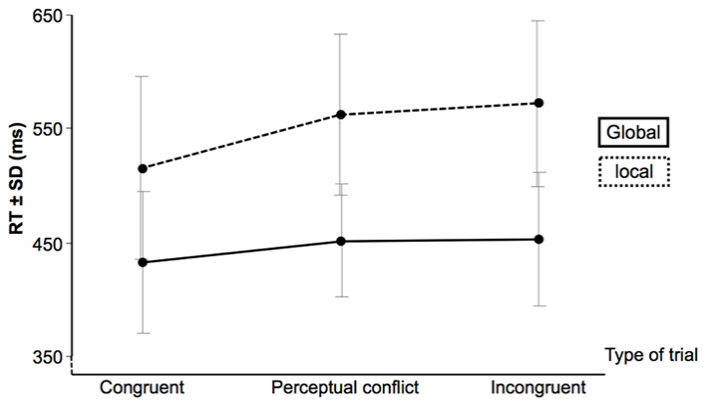
**Response time (RT) and standard deviation (SD) for correct answers**.

Participants were highly accurate in all experimental conditions (global level: 99.1 ± 1.8, 98.6 ± 1.9 and 95.4 ± 6.3% for congruent; with perceptual conflict and incongruent trials, respectively; local level: 99.1 ± 1.8, 96.1 ± 3.4 and 94.2 ± 4.2% for congruent; with perceptual conflict and incongruent trials, respectively). The GLM analysis on the RTs for correct answers indicated that the interaction between condition and type of trial was significant [*F*_(2, 10)_ = 9.5; *p* = 0.005; see Figure 3]. While participants were always faster to respond to global targets than for local targets (all *p*'s < 0.05 corrected for multiple comparisons), an interference effect was only present during local tasks (i.e., interference from the global unattended level): the congruent stimuli elicited faster RTs than conflicting perceptual and incongruent stimuli (*p* < 0.001). Thus, this interaction indicates a global-to-local interference effect.

### ERP results

**Figure 4:**
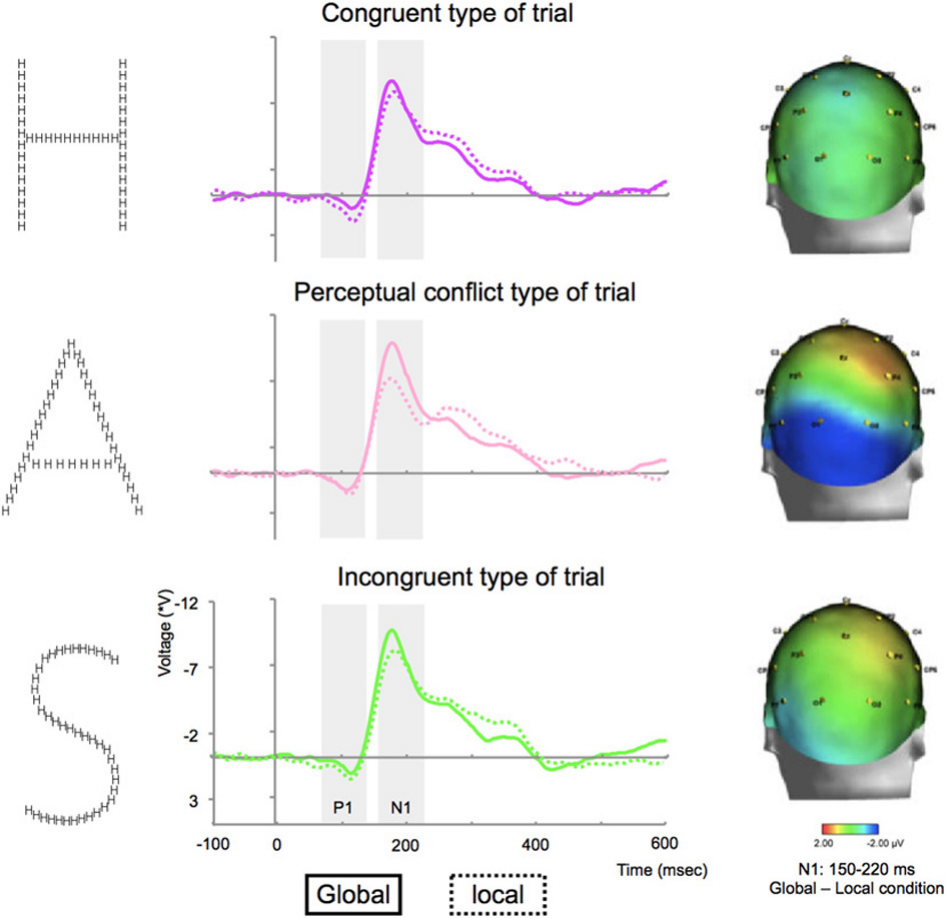
**Grand average ERP waveforms for congruent (top), with perceptual conflict (middle) and incongruent stimuli (bottom) on P7 electrodes and differences between the N1 component elicited by global and local tasks for congruent, with perceptual conflict and incongruent stimuli projected on a 3D scalp from a posterior head view**.

The statistical analysis of the mean amplitude of P1 on electrodes P7/P8 for correct trials did not show any interaction between condition x type of trial x side [*F*_(2, 10)_ = 1.5, *p* = 0.2] or between condition x type of trial [*F*_(2, 10)_ = 2.3, *p* = 0.1; Table 1, Figure 4].

**Table 1:** **Mean amplitudes for the different ERP components elicited on P7 and P8 electrodes according to the interference effect during the different tasks (± standard deviation)**.

**ERP component**	**Task**	**P7**			**P8**		
		**Congruent**	**Perceptual conflict**	**Incongruent**	**Congruent**	**Perceptual conflict**	**Incongruent**
P1: 80–130 ms	Global	0.5 ± 0.7	0.8 ± 1.2	0.6 ± 1.0	1.2 ± 1.2	2.0 ± 0.8	1.7 ± 0.9
	Local	1.6 ± 1.2	0.8 ± 1.1	1.7 ± 0.9	−0.3 ± 0.7	0.1 ± 0.8	−0.01 ± 0.5
N1: 150–220 ms	Global	−6.8 ± 1.0	−7.7 ± 1.0	−7.4 ± 0.9	−7.8 ± 1.1	−8.5 ± 1.0	−8.2 ± 1.0
	Local	−3.5 ± 1.0	−2.2 ± 1.0	−0.5 ± 1.0	−5.8 ± 1.5	−6.8 ± 1.3	−6.0 ± 1.3

The statistical analysis of the mean amplitude of N1 on electrodes P7/P8 for correct trials did not show an interaction between condition × type of trial × side [*F*_(2, 10)_ = 2.3, *p* = 0.2]. The analysis revealed an interaction between condition × type of trial [*F*_(2, 10)_ = 6.1, *p* = 0.02; see Figure 4], characterized by a lack of difference between the amplitude elicited during global and local tasks for congruent stimuli (*p* > 0.05); while both conflicting perceptual and incongruent stimuli elicited greater N1 amplitude during global tasks compared to local tasks (*p*'s < 0.01, Table 1, Figure 4).

## Discussion

The present study is the first to document an interference effect occurring as early as the time range of the N1 component during global/local processing. As expected, the behavioral measures showed a classical GPE. We observed a global advantage for the detection of global targets as well as a global-to-local interference: the response time increased during the detection of a local target when the global level represents information differing from the local level. ERP revealed that when the same letter was present at the global and local levels, comparable N1 amplitude was elicited for both global and local tasks, whereas when the letter presented at the global level differed from the one present at the local level, the N1 amplitude was reduced during local processing (compared to the amplitude recorded during the global detection task). Note that this interference effect was not observed for the P1 amplitude. Together, the present results suggest an interference effect on early steps of visual processing.

Many previous studies did not show any N1 amplitude differences between global and local processing due to interference effects (Heinze and Munte, 1993; Han et al., 1997, 1999, 2001; Evans et al., 2000; Yoshida et al., 2007). We hypothesized that this lack of difference comes from the fact that these studies have used only stimuli with perceptual conflict (named “neutral” situations in these studies) and incongruent compound stimuli (Evans et al., 2000; Yoshida et al., 2007), or too few congruent trials that could have reduced the interference effect (numerous trials with no target; Heinze and Munte, 1993; Han et al., 1997, 1999, 2000a, 2001). In stimuli with perceptual conflict and in incongruent stimuli, the target letter at the local level differs from the letter present at the global level, leading to potential perceptual and semantic conflicts (Weissman et al., 2003). In this vein, it was suggested that interference effects are initiated as soon as different identifiable stimuli are present at the global and the local levels (e.g., Poirel et al., 2006, 2008; Beaucousin et al., 2011). Specifically regarding incongruent stimuli, when the distractor present at the irrelevant global level is also a target letter, a conflict is induced that may have to be inhibited, resulting in a motor response conflict. For stimuli with perceptual conflict, even if the distractor at the global level is not a target letter, this information must be inhibited to correctly perform the local level (see Poirel et al., 2008, Experiment 2).

In our previous work, we suggested that the reduced amplitude of N1 during local tasks reflected an automatic attentional capture from the global level information, leaving fewer attentional resources for processing the local information (Beaucousin et al., 2011). The results from the present study, in which we presented three types of stimuli (i.e., congruent, with perceptual conflict and incongruent), corroborate the previous assumption that when a perceptual conflict is present (as compared to a non-conflicting congruent situation) the amplitude of N1 differs between global and local tasks. Therefore, the N1 amplitude modulation could be considered as a reflection of an interference from global information during local processing when conflicting stimuli are presented, rather than an indicator of differences between global and local processing as was previously suggested (Han et al., 1997, 1999, 2000a,b, 2001, 2003; Proverbio et al., 1998; Evans et al., 2000; Jiang and Han, 2005; Han and Jiang, 2006; Conci et al., 2011). This interpretation is in agreement with biological brain models of visual recognition (see Bullier, 2001; Hedgé, 2008). For example, in the “coarse-to-fine” model, the global information conveyed by rapid magnocellular visual channels allows for an initial perceptual analysis of visual inputs, which then guides the subsequent analysis of local information conveyed by slow parvocellular visual channels through feedback signals into low-level areas (such as the primary visual cortex, Bullier, 2001; see also Peyrin et al., 2003, 2010). This fast global processing could be conveyed within the brain's ventral pathway and would be able to guide—or interfere with—the subsequent more detailed and slower local processing of the visual scene (Fabre-Thorpe, 2011). The present findings could also fit with the view that fast magnocellular projections (that preferentially conveyed global information, see Hedgé, 2008; Boeschoten et al., 2005) could initially activate the orbitofrontal cortex, that initiates recognition mechanisms, back-projecting to the inferior temporal cortex to influence in a top-down manner the subsequent processing of local information (Kveraga et al., 2007, see also Bar et al., 2006). Such processing strengthen the assumption that interference between global and local processes effects could occurs as soon as global and local levels represent different information, as it is the case in conflicting perceptual and incongruent stimuli in which two different letters are presented at the two levels. Regarding these models, the present study shows for the first time that an interference effect from the global information level occurred very fast—approximately 150 ms—most likely in the extra-striate cortex where the N1 component is thought to be generated (Di Russo et al., 2002). In conclusion, the present results suggest that the human brain is already sensitive to “the forest before the trees” feeling (Navon, 1977) in the initial perceptual analysis of visual inputs.

### Conflict of interest statement

The authors declare that the research was conducted in the absence of any commercial or financial relationships that could be construed as a potential conflict of interest.
